# 596 The Introduction of Fish Skin Xenograft to a Burns Unit in a Country at War

**DOI:** 10.1093/jbcr/iraf019.225

**Published:** 2025-04-01

**Authors:** Steven Jeffery

**Affiliations:** Kyiv Burn Centre

## Abstract

**Introduction:**

Because of war there are very large numbers of big full thickness burns arriving at the country’s biggest burn centre.

Burn Surgeons in this country do not have access to allograft, and have little access to pig xenograft.

Fish skin xenograft is freeze-dried and does not require refrigeration.

**Methods:**

After appropriate training, Fish skin xenograft was introduced to the burn service.

**Results:**

The availability of fish xenograft allowed for larger burn excisions than prior to the intervention and allowed earlier autologous skin grafting.

**Conclusions:**

In the absence of allograft, fish skin xenograft allows for earlier coverage of large areas of full thickness burns.

**Applicability of Research to Practice:**

When considering what to donate to countries at war, priority should be given to the donation of fish skin xenograft.

**Funding for the Study:**

The mission to this country was funded by the British Red Cross.

The fish skin xenograft was donated by the manufacturers.

